# Double chambered right ventricle with severe calcification of the tricuspid valve in an elderly woman: a case report

**DOI:** 10.1186/1752-1947-5-210

**Published:** 2011-05-27

**Authors:** Nozomu Tamai, Shigenori Ito, Kotaro Morimoto, Masahiko Inomata, Takayuki Yoshida, Shin Suzuki, Yoshimasa Murakami, Koichi Sato

**Affiliations:** 1 Division of Cardiology, East Medical Center, Higashi Municipal Hospital, City of Nagoya; 1-2-23 Wakamizu, Chikusa-ku, Nagoya-shi, Aichi, 464-8547, Japan

## Abstract

**Introduction:**

Double chambered right ventricle is a rare congenital cardiac anomaly in which the right ventricle is divided into two chambers by an anomalous muscle bundle. The diagnosis of this disorder is difficult in adults. Calcification of the tricuspid valve is extremely rare, and very few cases have been reported. Most cases of tricuspid valve calcification had a congenital disorder with high pressure in the right ventricle.

**Case presentation:**

We report a rare case of a 71-year-old Japanese woman who presented with chest discomfort, and was found to have a double chambered right ventricle with severe calcification of the tricuspid valve. This abnormality was found by echocardiography, and the diagnosis was confirmed by multislice cardiac computerized tomography, cardiac magnetic resonance imaging, and cardiac catheterization. Our patient rejected surgical repair, and medical therapy with carvedilol was effective to reduce her symptoms.

**Conclusion:**

Calcification of the tricuspid valve is extremely rare, and considered to be due to high pressure in the right ventricle. To the best of our knowledge, there are no other reported cases of this combination of double chambered right ventricle and calcification of the tricuspid valve.

## Introduction

Double chambered right ventricle (DCRV) is a rare congenital cardiac anomaly in which the right ventricle is divided into two chambers of high pressure proximal and low pressure distal portion, by an anomalous muscle bundle. DCRV is described to be associated with different congenital disorders, most commonly with a membranous or malalignment type ventricular septal defect (VSD). An association with sub-aortic stenosis, pulmonary valve stenosis, atrial septal defect, double outlet right ventricle, and tetralogy of Fallot has also been reported [1]. Most patients with DCRV are diagnosed and repaired in childhood or adolescence; in contrast, the diagnosis is sometimes difficult in adults [2,3].

Calcification of the tricuspid valve is rare, and very few cases have been reported [4-7]. The mechanism of calcification of the tricuspid valve is considered to be due to the high pressure or volume overload of the right ventricle associated with congenital disorder.

## Case presentation

A 71-year-old Japanese woman was referred to our institution for further evaluation of chest discomfort, heavy dizziness and nausea after the use of nitroglycerin prescribed by a general practitioner.

On physical examination, cardiac auscultation revealed a systolic ejection murmur at her left sternal border. Her blood pressure was 130/60 mmHg, heart rate 68 beats/min, and her peripheral oxygen saturation was 98% in the room air. The 12-lead electrocardiogram revealed small negative T waves in leads III and a VF. A chest X-ray showed no pulmonary congestion, and the cardiothoracic ratio was 51%. Mild hypercholesterolemia was shown on her blood chemistry. Her B-type natriuretic peptide level was 81 pg/ml.

**Figure 1:**
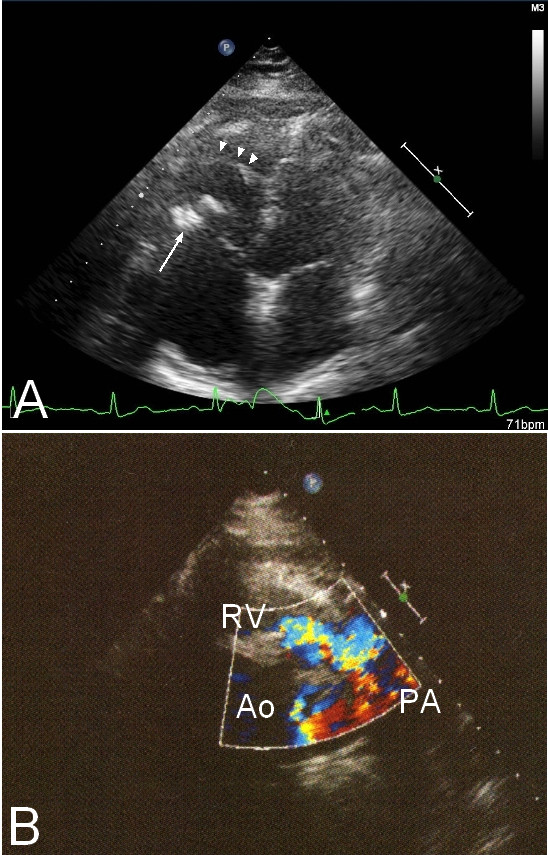
**Transthoracic echocardiography**. (A) Apical four chamber view shows anomalous muscle bundle (arrowheads) and calcification of tricuspid valve (arrow). (B) Color Doppler study from parasternal short axis view shows high velocity flow signal in RVOT.

Transthoracic echocardiography revealed right ventricular hypertrophy, and a calcified lesion around her tricuspid valve (Figure 1A). A Doppler study showed a high velocity flow signal in her right ventricular outflow tract (RVOT), and a mild tricuspid regurgitation (Figure 1B). Her tricuspid valve area was calculated to be 2.2 cm^2^.

**Figure 2:**
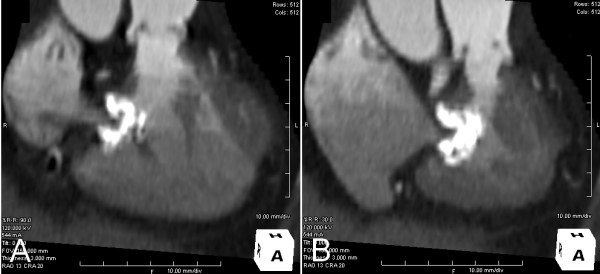
**Multislice cardiac enhanced CT: systolic RVOT narrowing is observed**. (A) Diastole. (B) Systole.

**Figure 3:**
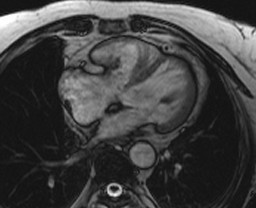
**Cardiac MRI shows no parenchymatous tissue or enhancement in the calcified lesion of the tricuspid valve**.

Multislice cardiac enhanced computerized tomography (CT) scanning showed an anomalous muscular bundle, dividing her right ventricle into two different compartments, which led to the RVOT stenosis. The stenosis was not obvious in the diastole, but especially severe in the systole. A calcification around her tricuspid valve, which seemed to be involved in the stenosis, was also observed (Figure 2). No calcification was observed around the other three valves. On the cardiac enhanced magnetic resonance imaging (MRI), the calcified lesion contained very little parenchymatous tissue and revealed no enhancement (Figure 3).

**Figure 4:**
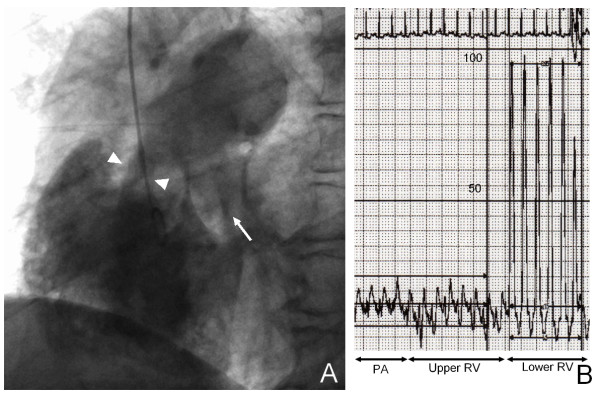
**Cardiac catheterization**. (A) Projection from right atrium shows RVOT stenosis (arrow heads) right to left flow through the patent foramen ovale. (B) Pullback pressure recordings demonstrated a pressure gradient of 74 mmHg across the RVOT stenosis.

A cardiac catheterization was performed. Her coronary angiography showed no abnormalities. Right to left flow through her patent foramen ovale, but no left to right shunt flow, was observed (Figure 4A). Her pulmonary artery pressure was 24/13 mmHg; the pullback pressure recordings demonstrated a pressure gradient of 74 mmHg across the RVOT stenosis, and her right ventricular pressure was 97/5 mmHg (Figure 4B). The diagnosis of double chambered right ventricle was confirmed based on the stated findings. Our patient refused surgical correction, and so beta-blockade (calvedilol) was prescribed. This was effective in reducing her symptoms after discharge.

## Discussion

DCRV is a rare congenital cardiac anomaly in which the right ventricle is divided into two chambers by anomalous muscle bundle. Most cases of DCRV are associated with different congenital disorders such as VSD, and the flow abnormalities related to these disorders are considered to be involved in the postnatal development of the proliferation of the muscle bundle. In our case, right to left flow through the patent foramen ovale was observed, but no left to right shunt flow (including VSD) was observed. Multislice cardiac enhance CT is usually recorded only at the diastole, but the stenosis became severe in the systole. Motion recordings of her right ventricle were useful for diagnosis.

A severe calcification of the tricuspid valve was observed in our case, but the other three valves were not calcified. There was no tricuspid stenosis and tricuspid regurgitation was mild. However, results from the CT scanning and MRI study suggested that this lesion was involved in the RVOT stenosis. Reports of calcification in the tricuspid valve are very rare [4-7]. The mechanism of calcification of the tricuspid valve is considered to be due to the high pressure or volume overload of the right ventricle associated with congenital disorder [7]. Most cases previously reported are associated with pulmonary stenosis [4,6], some cases are with atial septal defect [5,6], and one case was with rheumatic valve disease [7].

In our case, it is suspected that the use of nitroglycerin in RVOT stenosis led to low output and a decrease in systolic blood pressure; beta-blockade was effective to reduce systolic stenosis and also cardiac oxygen consumption.

## Conclusion

DCRV is a rare cardiac anomaly, and is difficult to diagnose in adults. Calcification of the tricuspid valve is also an extremely rare disorder, which is suspected to be associated with high pressure of the right ventricle. To the best of our knowledge, there is no other case report of this combination of DCRV and calcification of the tricuspid valve.

## Abbreviations

CT: computerized tomography; DCRV: double chambered right ventricle; MRI: magnetic resonance imaging; RVOT: right ventricular outflow tract; VSD: ventricular septal defect.

## Consent

Written informed consent was obtained from the patient for publication of this case report and any accompanying images. A copy of the written consent is available for review by the Editor-in-Chief of this journal.

## Competing interests

The authors declare that they have no competing interests.

## Authors' contributions

NT contributed to the management of the patient, and was a major contributor in writing the manuscript. KM, MI and SS analyzed and interpreted our patient data. TY analyzed cardiac enhanced multislice CT. SI, YM and KS were responsible for manuscript editing and advice on the literature review. All authors read and approved the final manuscript.
